# Ultraprotective versus apneic ventilation in acute respiratory distress syndrome patients with extracorporeal membrane oxygenation: a physiological study

**DOI:** 10.1186/s40560-022-00604-9

**Published:** 2022-03-07

**Authors:** Peter T. Graf, Christoph Boesing, Isabel Brumm, Jonas Biehler, Kei Wieland Müller, Manfred Thiel, Paolo Pelosi, Patricia R. M. Rocco, Thomas Luecke, Joerg Krebs

**Affiliations:** 1 Department of Anesthesiology and Critical Care Medicine, University Medical Centre Mannheim, Medical Faculty Mannheim of the University of Heidelberg, Theodor-Kutzer Ufer 1-3, 68167 Mannheim, Germany; 2 Institute for Computational Mechanics, Technical University Munich, Boltzmannstraße 15, 85748 Garching, Germany; 3 Ebenbuild GmbH, Schinkelstrasse 44, 80805 Munich, Germany; 4 Department of Surgical Sciences and Integrated Diagnostics, University of Genoa, Viale Benedetto XV 16, Genoa, Italy; 5 Anesthesiology and Critical Care, San Martino Policlinico Hospital, IRCCS for Oncology and Neurosciences, Genoa, Italy; 6 Laboratory of Pulmonary Investigation, Carlos Chagas Filho Institute of Biophysics, Federal University of Rio de Janeiro, Centro de Ciências da Saúde, Avenida Carlos Chagas Filho, 373, Bloco G-014, Ilha do Fundão, Rio de Janeiro, Brazil

**Keywords:** Acute respiratory distress syndrome, Ventilator-induced lung injury, Respiratory mechanics, Respiratory function, Mechanical ventilation, Extracorporeal membrane oxygenation, Strain, Transpulmonary pressure

## Abstract

**Background:**

Even an ultraprotective ventilation strategy in severe acute respiratory distress syndrome (ARDS) patients treated with extracorporeal membrane oxygenation (ECMO) might induce ventilator-induced lung injury and apneic ventilation with the sole application of positive end-expiratory pressure may, therefore, be an alternative ventilation strategy. We, therefore, compared the effects of ultraprotective ventilation with apneic ventilation on oxygenation, oxygen delivery, respiratory system mechanics, hemodynamics, strain, air distribution and recruitment of the lung parenchyma in ARDS patients with ECMO.

**Methods:**

In a prospective, monocentric physiological study, 24 patients with severe ARDS managed with ECMO were ventilated using ultraprotective ventilation (tidal volume 3 ml/kg of predicted body weight) with a fraction of inspired oxygen (FiO_2_) of 21%, 50% and 90%. Patients were then treated with apneic ventilation with analogous FiO_2_. The primary endpoint was the effect of the ventilation strategy on oxygenation and oxygen delivery. The secondary endpoints were mechanical power, stress, regional air distribution, lung recruitment and the resulting strain, evaluated by chest computed tomography, associated with the application of PEEP (apneic ventilation) and/or low *V*_T_ (ultraprotective ventilation).

**Results:**

Protective ventilation, compared to apneic ventilation, improved oxygenation (arterial partial pressure of oxygen, *p* < 0.001 with FiO_2_ of 50% and 90%) and reduced cardiac output. Both ventilation strategies preserved oxygen delivery independent of the FiO_2_. Protective ventilation increased driving pressure, stress, strain, mechanical power, as well as induced additional recruitment in the non-dependent lung compared to apneic ventilation.

**Conclusions:**

In patients with severe ARDS managed with ECMO, ultraprotective ventilation compared to apneic ventilation improved oxygenation, but increased stress, strain, and mechanical power. Apneic ventilation might be considered as one of the options in the initial phase of ECMO treatment in severe ARDS patients to facilitate lung rest and prevent ventilator-induced lung injury.

*Trial registration:* German Clinical Trials Register (DRKS00013967). Registered 02/09/2018. https://www.drks.de/drks_web/navigate.do?navigationId=trial.HTML&TRIAL_ID=DRKS00013967.

**Supplementary Information:**

The online version contains supplementary material available at 10.1186/s40560-022-00604-9.

## Background

Acute respiratory distress syndrome (ARDS) is a severe lung dysfunction due to inflammation, edema formation and pulmonary shunt resulting in hypoxia and often necessitating invasive mechanical ventilation (MV) [1]. In the most severe cases of ARDS with refractory hypoxemia, extracorporeal membrane oxygenation (ECMO) has been proposed as a viable therapy to maintain oxygenation and oxygen delivery (DO_2_) [2], possibly improving survival [3]. Although the use of ECMO in patients with ARDS is increasing [4], there is a paucity of physiological data regarding the optimal ventilator settings during ECMO treatment [5]. The Extracorporeal Life Support Organization (ELSO) recommends a ventilation strategy with low respiratory rates (RR), low inspiratory plateau pressure (*P*_plat_) and moderate levels of positive end-expiratory pressure (PEEP) to limit ventilator-induced lung injury (VILI) [6]. Since the main principle of MV during ECMO is to protect the lung, gas exchange should primarily be managed with ECMO [6].

MV during ECMO, even with ultraprotective tidal volume (*V*_T_), may induce VILI due to the transmitted energy by the ventilator resulting in increased stress (transpulmonary pressure at end-inspiration) and strain (*V*_T_/end-expiratory lung volume) [7, 8] as well as high fractions of inspired oxygen (FiO_2_) [9] in the inflamed pulmonary parenchyma.

As the optimal ventilation strategy in ECMO patients is insufficiently defined [10], the primary endpoint of this physiological, short-term study was to investigate the effects of ultraprotective compared to apneic ventilation on oxygenation and DO_2_ in ARDS patients treated with ECMO in the initial phase of the management. We further hypothesized whether apneic ventilation with the sole application of a low FiO_2_ and reasonable PEEP reduces cyclic lung volume changes and may better protect the lung parenchyma from VILI. Therefore, the secondary endpoints were to evaluate mechanical power, stress, regional air distribution, recruitment and the resulting strain associated with the application of PEEP (apneic ventilation) and/or low *V*_T_ (ultraprotective ventilation).

## Methods

The study was approved by the local ethics committee (Medizinische Ethikkommission II, University Medical Centre Mannheim, Medical Faculty Mannheim of the University of Heidelberg, Mannheim, registration number 2016-601 N-MA) and registered at the German Clinical Trials Register (DRKS00013967). After obtaining written informed consent we collected prospective data from 24 patients with severe ARDS managed with ECMO admitted to the Department of Anesthesiology and Critical Care Medicine, University Medical Centre Mannheim, Medical Faculty Mannheim of the University of Heidelberg in Mannheim, Germany. Detailed inclusion and exclusion criteria, as well as criteria for discontinuation of the study are provided in Additional file 1.

ECMO therapy was initiated as indicated by the attending physician in accordance with the current ELSO guidelines [6]. SAPS II [11], SOFA [12] and APACHE II scores [13] were calculated for each patient at ICU admission. The RESP [14] and PRESERVE scores [15] were calculated immediately before ECMO cannulation. Vascular access for the extracorporeal circuit was established with a 29 French drainage cannula (HLS Cannula, Maquet, Rastatt, Germany) in the femoral vein and a 21 or 23 French return cannula (HLS Cannula, Maquet, Rastatt, Germany) in the internal jugular vein. The ECMO circuit was driven by a magnetically levitated rotor pump (Centrimag Circulatory Support System, Abbot GmbH, Wiesbaden, Germany) and completed with a gas exchange membrane (PLS System, Maquet, Rastatt, Germany). Patients were sedated (Richmond Agitation-Sedation Score of − 5) and paralyzed throughout each measurement [16]. Norepinephrine was used if mean arterial pressure (MAP) was below 65 mmHg despite sufficient intravascular volume. Dobutamine was used if cardiac index was below 2.0 l/min/m^2^ despite sufficient cardiac pre- and afterload. Extracorporeal blood flow was titrated to achieve an arterial partial pressure of oxygen (PaO_2_) of at least 60 mmHg with ventilation settings (Engström Carescape™, GE Healthcare, Munich, Germany) chosen by the attending physician. ECMO gas flow was titrated to achieve an arterial pH of 7.35 to 7.45. For the measurement of esophageal pressure, an esophageal balloon catheter (NutriVent™, Sidam Biomedical Solutions, Mirandola, Italy) was inserted, filled with 2.5 ml of air as indicated by the manufacturer and connected to the ventilator. Catheter position was confirmed in all patients as previously described [17]. Airway and esophageal pressures were recorded during end-expiratory and end-inspiratory hold (5 s without gas flow from the ventilator), respectively [18]. Cardiac output (CO) was measured using a thermodilution catheter (4F/5F Pulsiocath™, Pulsion Medical Systems, Feldkirchen, Germany) and a CO monitor (PiCCOplus™, Pulsion Medical Systems, Munich, Germany). For intermittent blood gas analyses for the measurement of pH, the PaO_2_ and carbon dioxide (PaCO_2_), the arterial oxygen saturation (SaO_2_) and hemoglobin (Hb) a blood gas analyzer (Radiometer ABL 800 FlexQ, Radiometer GmbH, Krefeld, Germany) was used.

### Study protocol

After ensuring adequate analgosedation and neuromuscular blockade, dynamic recruitment was performed by slowly increasing the PEEP set by the attending physician from the individual baseline to 35 cmH_2_O over a time period of 5 min. The difference between end-inspiratory and end-expiratory airway pressure was kept at 15 cmH_2_O in a pressure-controlled ventilation mode. After 2 min of dynamic recruitment at a *P*_plat_ of 50 cm H_2_O, PEEP was empirically set to 30 cm H_2_O and a standardized ventilation setting (ultraprotective ventilation, RR of 12/min, *V*_T_ of 3 ml/kg IBW, inspiration-to-expiration ratio of 1:1) established. PEEP was then decreased stepwise by 2 cm H_2_O and after a 10-min equilibration period we calculated the static elastance of the respiratory system (*E*_stat,RS_) as driving pressure (*P*_driv_)/*V*_T_. The decremental PEEP trial was stopped if *E*_stat,RS_ did not progressively decrease because of the reduction of PEEP. For an unequivocal identification of the lowest achievable individual *E*_stat,RS_ of the patient, a further reduction of PEEP had to result in a marked increase in *E*_stat,RS_. Subsequently, another recruitment maneuver was performed, and PEEP was set to the lowest *E*_stat,RS_ [18]. Without further modifications to the established ventilation settings, the fraction of inspired oxygen was adjusted to 21%. After another 30-min equilibration period respiratory mechanic and hemodynamic parameters were assessed. Blood gas samples from an arterial catheter were analyzed. These measurements were then repeated with a FiO_2_ of 50% and 90% with a 30-min equilibration period between each measurement. Subsequently, the ventilation mode was changed from ultraprotective ventilation to apneic ventilation without any changes in PEEP and the measurements were repeated at a FiO_2_ of 21%, 50% and 90%, respectively. During each measurement respiratory mechanic and hemodynamic parameters were assessed. Blood gas samples from an arterial catheter were analyzed. Neither ECMO blood nor gas flow were changed during the study period. Further details of the ventilator management are provided in the Additional file 1. Ventilation mode was then again changed to ultraprotective ventilation with a RR of 12/min, a *V*_T_ of 3 ml/kg IBW, an inspiration-to-expiration ratio of 1:1 and unchanged PEEP and the patients transferred to the CT scanner. Images of the whole lungs were acquired at end-inspiratory hold, at end-expiratory hold at PEEP as well as at a PEEP of 0 mmHg (ZEEP). We used a second-generation dual source CT scanner (Somatom Definition Flash) with 32 × 0.6 mm collimation, 89/76 reference mAs at 120 kV, a pitch of 0.8 and 0.5 s rotation time. CT scans were first segmented automatically with the Medical Imaging Interaction Toolkit (Version 2018.04.2, https://www.mitk.org/). The resulting segmentations were then used as a basis for manual segmentation to differentiate nonaerated lung and extrapulmonary soft tissue. Additional file 1: Fig. S1 shows the schematic flow chart of the study design.

### Calculations

Gas exchange, respiratory system mechanics and hemodynamics were calculated after an equilibration period for each ventilation strategy and fraction of inspired oxygen (see Additional file 1). Lung parenchyma aeration was classified following the conventional thresholds [19] in the CT scans and segmented into a non-dependent and dependent compartment along a horizontal plane through the tracheal bifurcation using in-house software (details are provided in Additional file 1). Recruitment due to PEEP and *V*_T_ as well as static and dynamic strain were calculated as described in Additional file 1.

### Statistical analysis

The sample size calculation for testing the primary hypothesis (PaO_2_ would be decreased in apneic ventilation compared to ultraprotective ventilation with a FiO_2_ of 50%) was based on data obtained from 5 patients, not included in the study. Accordingly, we expected that a sample size of 24 would provide the appropriate power (1 – *β* = 0.9) to identify significant (*α* = 0.05) differences considering a partial *η*^2^ of 0.11 and an effect size of 0.35 analyzed with a repeated measurement ANOVA. Power analysis was performed with G*Power 3.1.9.7. For continuous variables, the normality of the data and the homogeneity of variances were tested by means of the Shapiro–Wilk test and Levene’s median test, respectively. As per the study protocol, longitudinal physiological and CT data were analyzed using repeated measures ANOVA followed by Holm–Sidak’s post-hoc test or the Friedman procedure as appropriate to control for variability between patients. Single timepoint data were analyzed using one-way ANOVA followed by Holm–Sidak’s post-hoc test or Mann–Whitney test, as appropriate. The results are expressed as mean ± standard deviation. The level of significance was set at *p* < 0.05. Statistical analysis was performed using SigmaPlot 12.5 (Systat Software GmbH, Erkrath, Germany).

## Results

### Patient characteristics

Twenty-four patients with severe ARDS treated with veno-venous ECMO completed the study and were included in the analysis. Table 1 shows the demographic and clinical characteristics of the patients. Nineteen patients had a primary pulmonary, while five patients had an extrapulmonary cause of ARDS. At study inclusion, patients were receiving MV for a mean duration of 5.7 ± 3.5 days. The mean duration of ECMO support in the study cohort was 13.5 ± 6.0 days with an ICU mortality of 42%.

**Table 1:** Anthropometric characteristics of the patients included in the study

	*n* = 24
Age [years]	57.0 ± 9.5
Male sex [%]	67
Body mass index [kg/m^2^]	32.9 ± 7.7
MV before study [days]	5.5 ± 3.5
Cause of ARDS	
Pulmonary [%]	79
Extrapulmonary [%]	21
SAPS II	70.1 ± 11.6
SOFA	14.2 ± 3.3
APACHE II	30.8 ± 7.1
RESP score	− 5.1 ± 4.3
PRESERVE score	5.5 ± 2.1
Duration ECMO support [days]	13.5 ± 6.0
Length of ICU stay [days]	39.3 ± 20.5
ICU Mortality [%]	42

Data are reported as mean ± sd or percentage as appropriate

*MV* mechanical ventilation, *SAPS II* Simplified Acute Physiology Score II, *SOFA* Sequential Organ Failure Assessment, *APACHE II* Acute Physiology and Chronic Health Evaluation II, *RESP* Respiratory ECMO Survival Prediction, *PRESERVE* Predicting Death for Severe ARDS on vv-ECMO; *ICU* intensive care unit

### Effect on oxygenation and oxygen delivery

**Fig. 1:**
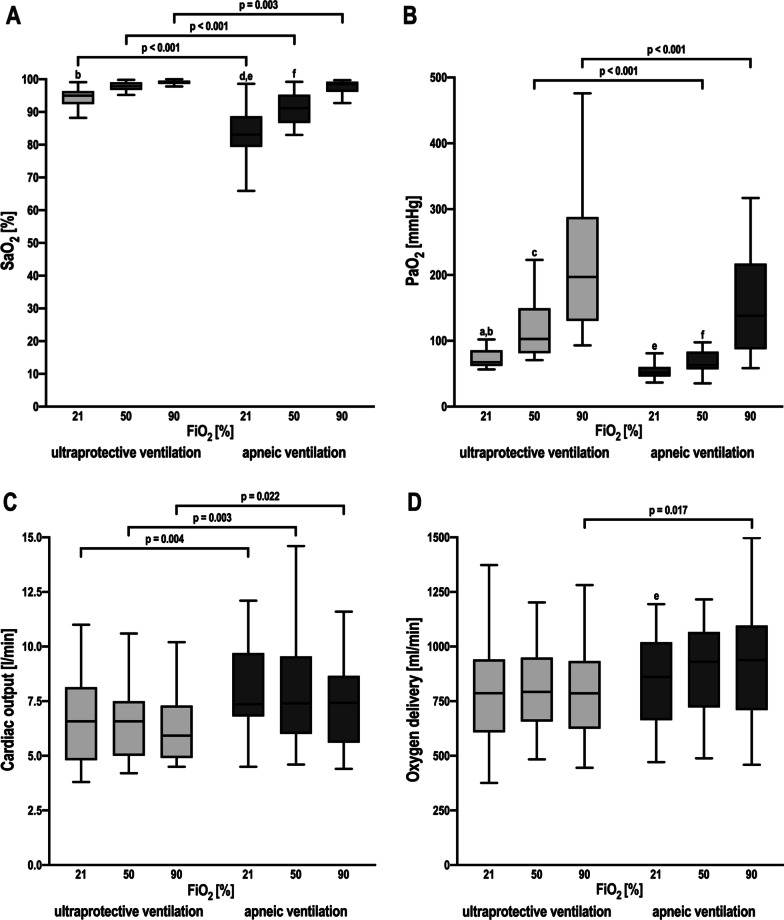
Oxygenation and oxygen delivery for ultraprotective and apneic ventilation and three different fractions of inspired oxygen. **A** Arterial oxygen saturation; SaO_2_, arterial oxygen saturation, FiO_2_, fraction of inspired oxygen. **B** Arterial partial pressure of oxygen. PaO_2_, arterial partial pressure of oxygen, FiO_2_, fraction of inspired oxygen. **C** Cardiac output, FiO_2_, fraction of inspired oxygen. **D** Oxygen delivery. FiO_2_, fraction of inspired oxygen**,** brackets denote statistically significant differences between ventilation strategies, *p*-values are shown above the brackets. a: *p* < 0.05 ultraprotective ventilation with a FiO_2_ of 21% vs. ultraprotective ventilation with a FiO_2_ of 50%; b: *p* < 0.05 ultraprotective ventilation with a FiO_2_ of 21% vs. ultraprotective ventilation with a FiO_2_ of 90%; c: *p* < 0.05 ultraprotective ventilation with a FiO_2_ of 50% vs. ultraprotective ventilation; with a FiO_2_ of 90%; d: *p* < 0.05 apneic ventilation with a FiO_2_ of 21% vs. apneic ventilation with a FiO_2_ of 50%; e: *p* < 0.05 apneic ventilation with a FiO_2_ of 21% vs. apneic ventilation with a FiO_2_ of 90%; f: *p* < 0.05 apneic ventilation with a FiO_2_ of 50% vs. apneic ventilation with a FiO_2_ of 90%

SaO_2_ significantly increased from a FiO_2_ of 21% to 90% in ultraprotective ventilation and at every FiO_2_ step in apneic ventilation and was uniformly higher in ultraprotective ventilation than in apneic ventilation at each FiO_2_ (Fig. 1A). PaO_2_ increased at each FiO_2_ step in ultraprotective ventilation and from 21 to 90% and 50% to 90% in apneic ventilation. PaO_2_ differed significantly between ultraprotective and apneic ventilation only when applying a FiO_2_ of 50% and 90% (Fig. 1B). CO was lower in ultraprotective compared to apneic ventilation (Fig. 1C). DO_2_ was higher for apneic ventilation with a FiO_2_ of 90%, compared to the corresponding ultraprotective ventilation (Fig. 1D).

### Effect on lung mechanics, hemodynamics and carbon dioxide elimination

Since lung mechanics, hemodynamics and carbon dioxide elimination did not differ between a FiO_2_ of 21%, 50% and 90%, we opted to present the pooled data during ultraprotective and apneic ventilation in Table 2.

**Table 2:** Physiological data during ultraprotective and apneic ventilation in severe ARDS patients treated with ECMO

	Ultraprotective ventilation	Apneic ventilation	*p*-values
PEEP [cm H_2_O]	15.4 ± 4.8	15.4 ± 4.8	*p* = 1.000
*P*_plat_ [cm H_2_O]	27.3 ± 6.4	15.4 ± 4.8	***p* < 0.001**
*P*_mean_ [cm H_2_O]	20.6 ± 5.0	15.4 ± 4.8	***p* < 0.001**
*P*_driv_ [cm H_2_O]	11.9 ± 5.8	0.0 ± 0.0	***p* < 0.001**
*E*_stat,RS_ [cm H_2_O/l]	29.1 ± 18.6	Not applicable	
*E*_stat,L_ [cm H_2_O/l]	22.2 ± 17.9	Not applicable	
*E*_stat,CW_ [cm H_2_O/l]	7.8 ± 3.1	Not applicable	
Stress [cm H_2_O]	8.2 ± 5.2	− 0.5 ± 3.7	***p* < 0.001**
Mechanical power [joule/min]	12.9 ± 3.8	not applicable	
ECMO blood flow [l/min]	4.0 ± 0.8	4.0 ± 0.8	*p* = 0.783
ECMO gas flow [l/min]	4.0 ± 1.2	4.0 ± 1.1	*p* = 0.811
PaCO_2_ [mmHg]	41.9 ± 6.7	53.8 ± 9.2	***p* < 0.001**
pHa	7.4 ± 0.1	7.3 ± 0.1	***p* < 0.001**
HR (beats/min)	93.0 ± 20.8	93.0 ± 17.6	*p* = 0.993
MAP [mmHg]	83.3 ± 12.9	83.3 ± 15.1	*p* = 1.000
CVP [mmHg]	15.8 ± 5.0	15.1 ± 3.1	*p* = 0.297
Noradrenaline [µg/kg/min]	0.2 ± 0.3	0.2 ± 0.3	*p* = 0.847
Dobutamine [µg/kg/min]	0.8 ± 2.1	0.8 ± 2.1	*p* = 0.859

Pooled physiological data from ultraprotective, respectively, apneic ventilation with a FiO_2_ of 21%, 50% and 90%

*p*-values < 0.05 are considered significant (repeated measurement ANOVA followed by Holm-Sidak’s post-hoc test)

Data are presented as mean ± standard deviation

*PEEP* positive end-expiratory pressure, *P*_*plat*_ plateau airway pressure, *P*_*mean*_ mean airway pressure, *P*_*driv*_ difference between end-inspiratory and end-expiratory tracheal pressure, *E*_*stat,Rs*_ static elastance of the respiratory system, *E*_*stat,L*_ static elastance of the lung, *E*_*stat,Cw*_ static elastance of the chest wall, *ECMO* extracorporeal membrane oxygenation, *PaCO*_*2*_ arterial partial pressure of carbon dioxide, *pHa* arterial pH, *HR* heart rate, *MAP* mean arterial pressure, *CVP* central venous pressure

*P*_plat_, *P*_mean_, *P*_driv_, stress and mechanical power were more reduced in apneic compared to ultraprotective ventilation with no changes in PEEP level. pH was lower during apneic ventilation compared to ultraprotective ventilation due to increased PaCO_2_.

### Air distribution in lung parenchyma, percentage of lung recruitment and strain

**Fig. 2:**
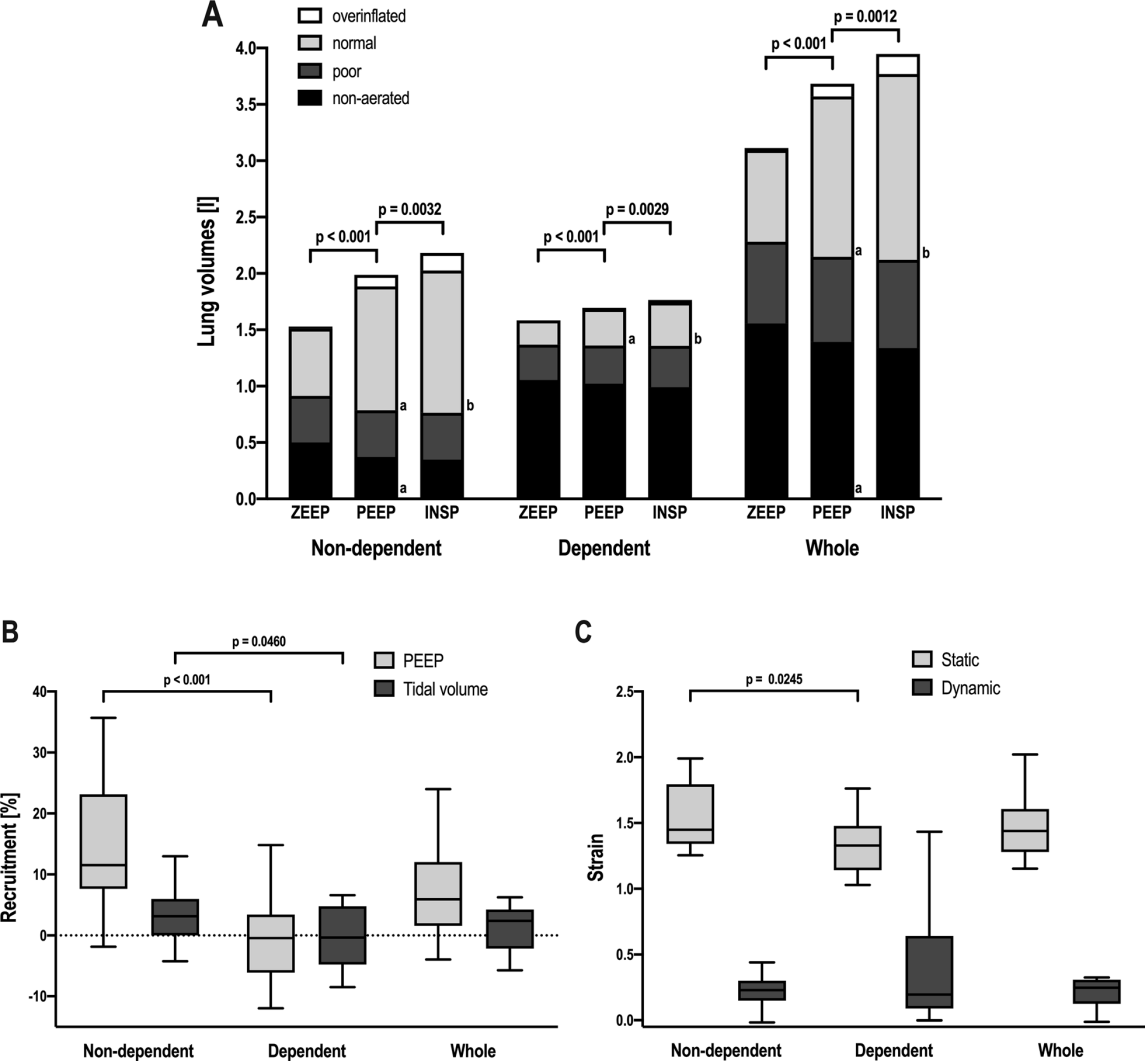
Air distribution in lung parenchyma, percentage of lung recruitment and strain in the non-dependent, dependent, and whole lung. **A** Air distribution in lung parenchyma in in the non-dependent, dependent, and whole lung. Black, non-aerated lung volume (− 100 to + 100 hounsfield units); dark grey, poorly inflated lung volume (− 500 to − 100 hounsfield units); light grey, normally inflated lung volume (− 501 to − 900 hounsfield units); white, overinflated lung volume (< − 900 hounsfield units), ZEEP, air distribution at zero end-expiratory pressure; PEEP, air distribution at end-expiratory hold, INSP, air distribution at end-inspiratory hold. **B** Percentage of recruitment associated to the application of apneic and ultraprotective ventilation in the non-dependent, dependent and whole lung. PEEP, recruitment due to positive end-expiratory pressure from zero positive end-expiratory pressure (ZEEP); tidal volume; recruitment due to tidal volume from end-expiratory pressure. **C** Static and dynamic strain in the non-dependent, dependent and whole lung. Static, static strain due to the application of PEEP; dynamic, dynamic strain due to the application of tidal volume. Brackets denote statistically significant differences, *p* values are shown above the brackets. a: *p* < 0.05 PEEP vs. ZEEP. **b** *p* < 0.05 INSP vs. PEEP

Figure 2 shows the air distribution in the lung parenchyma based on chest CT-scan analysis. Aerated lung volume increased with PEEP and *V*_T_. In the non-dependent lung segments as well as in the whole lung, application of PEEP significantly reduced the non-aerated lung volume compared to ZEEP. No additional reduction of non-aerated lung volume was obtained by the application of *V*_T_. The application of PEEP significantly increased the amount of normally aerated lung parenchyma compared to ZEEP in all lung segments. *V*_T_ further increased the normally aerated lung volume compared to PEEP (Fig. 2A). PEEP and *V*_T_ recruited significantly more lung volume in the non-dependent compared to the dependent part of the lung (Fig. 2B). Static strain was significantly higher in the non-dependent than in the dependent lung segments (Fig. 2C).

## Discussion

In patients with severe ARDS during ECMO treatment, we investigated the effects of *V*_T_, PEEP, and FiO_2_ on oxygenation as well as DO_2_ during an ultraprotective and an apneic ventilation strategy. As the transmitted energy by the ventilator to the lungs may be of critical importance to determine VILI, we also measured *P*_driv_, strain, stress and mechanical power to relate the physiological "cost" of the ventilation strategy to the potential benefit regarding oxygenation and DO_2_. We found that: (1) ultraprotective ventilation increased PaO_2_ and SaO_2_ but reduced CO compared to apneic ventilation; (2) both ventilation strategies preserved pH, PaCO_2_ and DO_2_, independently of the FiO_2_ without any changes in ECMO blood or gas flow; (3) *V*_T_ of 3 ml/kg IBW increased *P*_driv_, stress, strain and mechanical power compared to apneic ventilation; (4) PEEP as well as *V*_T_ recruited lung volume primarily in the non-dependent lung.

### Oxygenation and oxygen delivery

The application of an ultraprotective *V*_T_ resulted in higher SaO_2_ and PaO_2_ compared to the application of PEEP alone with an increase similar to that obtained with higher FiO_2_ in apneic ventilation (Fig. 1A, B). This may be the result of the higher airway pressures inducing parenchymal recruitment as the ultraprotective ventilation strategy increased the aerated lung volume in comparison to apneic ventilation (Fig. 2A). However, ultraprotective ventilation resulted in a significantly decreased CO, possibly due to the increased airway pressures compared to apneic ventilation (Fig. 1C). In severe ARDS, high *V*_T_ and airway pressures may affect CO by decreasing systemic venous return and increasing right ventricular outflow impedance [20]. As CVP does not significantly increase, our data implies, that in patients with most severe ARDS even a *V*_T_ of 3 ml/kg IBW and the resulting *P*_plat_ and *P*_driv_ negatively affects right ventricular afterload and thus CO. Although the apneic ventilation strategy preserved CO and thus DO_2_ compared to ultraprotective ventilation, we observed a significant decrease in SaO_2_ (Fig. 1D) especially at FiO_2_ of 21%, presumably because of the lower *P*_mean_ resulting in less aerated lung parenchyma [21]. On the other hand, as long as hemoglobin content and CO remain adequate, SaO_2_ levels as low as 80% might be physiologically acceptable [22]. Of note, as the ratio between ECMO flow and CO decreased in apneic ventilation compared to ultraprotective ventilation, the improvement in arterial oxygenation may not only be the result of the pulmonary recruitment but rather a result of the altered hemodynamics [22]. A key component of ECMO treatment is to increase or preserve DO_2_ [6, 22]. The safe threshold of DO_2_ for the individual patient is unknown, but a range between 400 and 600 ml/min/m^2^ is probably reasonable [23, 24] in critical ill patients. Therefore, both ventilation strategies appear to be viable in patients treated with ECMO. Because a further increase of DO_2_ due to higher *V*_T_ and concomitant tidal recruitment is not beneficial [24], using the ventilation strategy with the lowest energy transmission on lung parenchyma may be favorable to improve outcomes in these patients [7, 25].

### Respiratory system mechanics

Limiting *V*_T_ to approximately 6 ml/kg IBW and thus cyclic end-inspiratory hyperinflation of the inhomogeneous lung has been shown to reduce mortality and is recommended in all current guidelines for the management of ARDS [26, 27]. On the other hand, there is a subgroup of patients with most severe ARDS characterized by low end-expiratory lung volume and low recruitability of atelectatic lung parenchyma, that show tidal hyperinflation in spite of low *V*_T_ [28]. ECMO as well as extracorporeal carbon dioxide elimination may facilitate even lower *V*_T_ ventilation in these patients [29, 30]. Most medium-to-high-volume centers currently limit *V*_T_ and airway pressure in patients managed with ECMO [5] as “lung rest” to prevent VILI [31]. On the other hand, only 27% of these centers have an explicit MV protocol [31], which may be due to the fact that there is a paucity of physiological data describing the immediate physiological effects of different ventilation strategies during ECMO. In an international multicenter prospective cohort, Schmidt et al. reported a rather moderate reduction of *V*_T_ from 6.4 ± 2.0 to 3.7 ± 2.0 ml, of the RR from 26 ± 8 to 14 ± 6 breaths per minute and of *P*_driv_ from 20 ± 7 to 14 ± 4 cm H_2_O after the initiation of ECMO [5]. Of note, none of these centers utilized apneic ventilation as a first line ventilation strategy. As shown in the present study, ultraprotective ventilation strategy using even lower *V*_T_ (tidal volume 3 ml/kg ideal body weight) and RR induced a considerable *P*_driv_, which has been independently linked with mortality in ARDS patients during ECMO support [7]. In a recent study in ARDS patients treated with ECMO, Del Sorbo et al. demonstrated a linear relationship between inspiratory pressures, the resulting mechanical power and biomarkers of systemic inflammation [32]. Despite using an ultraprotective *V*_T_ of 2.4 ml/kg, there was substantial risk for biotrauma and ultimately VILI in patients with low respiratory system compliance. The authors concluded that the use of apneic ventilation may be favorable in these patients. Rozencwajg et al. tested three different “ultraprotective” strategies of MV with reduced plateau and driving pressure and consecutively *V*_T_. They found a significantly limited pulmonary biotrauma irrespectively of the tested strategy [33]. This is in accordance with the results of a large animal study were ventilation with limited driving pressure, low respiratory rate and thus limited mechanical power decreased lung injury in comparison with a ventilation strategy with higher driving pressures and higher minute ventilation [34].

### Air distribution, recruitment and strain

Locoregional excess of overinflation and alveolar cycling in inhomogeneous lung parenchyma are considered as pivotal factors causing VILI and are quantified by *P*_driv_, stress, strain and transmitted mechanical power [8]. Subsequently, a ventilation strategy for patients with most severe ARDS treated with ECMO should minimize these variables and simultaneously preserve cardiopulmonary function [10]. Therefore, the most “protective” ventilation strategy at least in theory should provide total "lung rest" by the sole application of an adequate level of PEEP, while carbon dioxide is removed through the ECMO membrane [35]. As higher PEEP levels during the first 3 days on ECMO support were independently associated with improved survival [36], the strategies to optimize PEEP warrant careful consideration. In our study, the application of PEEP titrated to the lowest *E*_stat,RS_ was associated with a reduced atelectatic lung volume in the non-dependent lung and increased aerated lung volume in both lung regions compared to ZEEP (Fig. 2A). On the other hand, this PEEP titration strategy was not able to recruit dependent lung segments, as shown in Fig. 2B. As shown previously PEEP titrated to the lowest *E*_stat,RS_ might be associated with negative end-expiratory transpulmonary pressure [18]. Aiming for a positive end-expiratory transpulmonary pressure might, therefore, be a viable strategy to further reduce atelectatic lung volume especially in dependent lung regions to reduce cyclic opening and closing of the alveoli (atelectrauma), to homogenize *V*_T_ distribution and to avoid overdistension [37]. The *V*_T_ of the ultraprotective ventilation strategy causes additional dynamic strain compared to the static strain induced by PEEP in the inflamed inhomogeneous lung. This resulted in a total mechanical power of approximately 13 J/min transmitted to the lung despite using a ventilation strategy with lower *P*_driv_ than described in two recent studies comparing MV during ECMO [10, 38]. Dynamic strain has been shown to be a more potent inductor of VILI compared to static strain resulting in pulmonary inflammation, edema formation and an increased mortality [39]. It is debatable whether a further reduction of *V*_T_ or the prevention of dynamic strain altogether would be more beneficial for the patient as the short- and long-term biological consequences of dynamic strain are incompletely understood. Of note, a recent trial by McNamee et al. found no statistically significant reduction in mortality when utilizing extracorporeal carbon dioxide removal devices to reduce *V*_T_ [40].

On the other hand, there is a paucity of data regarding the long-term consequences of omitting dynamic strain using an apneic ventilation strategy for a prolonged period of time [32].

### Clinical implications

Our data suggests that apneic ventilation might be a viable option in the initial phase of ECMO treatment in severe ARDS. Apneic ventilation preserved DO_2_ and reduced stress, strain and mechanical power transmitted to the lung compared with ultraprotective ventilation. According to current recommendations and clinical practice in medium-to-high-volume ECMO centers, the goal of ECMO treatment is to increase or preserve DO_2_ while minimizing VILI and prevent iatrogenic harm, especially in the initial phase of ARDS, while lung parenchyma is most vulnerable [5, 6, 31].

With resolving lung dysfunction and hemodynamic stabilization, spontaneous breathing should be considered to prevent diaphragmatic dysfunction [41] and allow ECMO weaning [6]. However, limited data is available regarding the benefits of spontaneous breathing in ARDS patients treated with ECMO and the potential self-inflicted lung injury [25]. Patients in the early phase of ARDS, characterized by low respiratory system compliance and low end-expiratory lung volume, are particularly susceptible to injurious transpulmonary pressure swings [42, 43] and poor patient–ventilator synchrony [44, 45]. This might lead to progression of lung inflammation and damage [42, 43, 46], highlighted in the concept of patient self-inflicted lung injury [47, 48]. Further studies are needed to investigate the optimal balance between spontaneous breathing and avoiding VILI in ARDS patients during ECMO [25].

### Limitations

There are some limitations in our study that should be addressed. First, we studied the physiological effects of apneic ventilation on oxygenation, DO_2_ and lung stress and strain in the initial phase of ECMO with deeply sedated and paralyzed patients. Therefore, the results of our study apply only to the early treatment phase without spontaneous breathing and with moderate to heavy sedation as recommended by current ELSO guidelines [6]. Neuromuscular blockade was used during the early treatment phase to prevent spontaneous breathing and to minimize airway pressures and transpulmonary pressure swings. This approach is supported by the experimental data of Guldner et al. [46] and clinical data published by Schmidt et al., where higher spontaneous respiratory rates during the first 2 days of ECMO were associated with higher 6-month mortality [5]. During ECMO treatment, discontinuation of neuromuscular blockade to allow spontaneous breathing is required to wean patients from the ECMO circuit. However, the optimal timing to minimize sedation and promote spontaneous breathing in the different phases of ARDS is unclear and needs further investigation [25].

Second, we studied the complex interaction between ventilation strategy, gas-exchange and hemodynamics only in a limited time frame. It is possible that the physiological effects of the ventilation strategy may vary during a prolonged observation period. The lower *P*_mean_ in apneic ventilation might promote better drainage of pulmonary interstitial fluid [49]. In contrast, cyclic lung stretch might be important to stimulate surfactant production [50]. Further studies are needed to investigate the long-term consequences of an apneic ventilation strategy and the effects on lung function and healing. As experimentally shown by Kolobow et al. decades ago, apneic ventilation with inadequate PEEP may decrease the functional residual capacity by approximately 50% if sustained for longer periods of time [35]. This may also have affected the data on lung aeration as the CT scan was performed after the comparison of different ventilation strategies. In case of a decreased end-expiratory lung volume, higher ECMO blood flows might be needed to maintain oxygenation despite the resulting “permissive” atelectasis and to protect the right ventricle from volume overload due to pulmonary hypertension. To test the hypothesis generated by our study, prospective trials with patient-centered outcomes are needed.

Third, this is a monocentric study in a specific population of patients with mainly pulmonary ARDS reflecting the specific management standard operating procedure and ventilation protocol of our unit. Therefore, the results may not be generalized to other ARDS subgroups treated with ECMO as there is data supporting the presence of at least two phenotypes in ARDS which might warrant different treatment strategies [51, 52, 53].

Fourth, only one single ventilation setting was investigated and compared to apneic ventilation. Thus, we cannot exclude that the effect of the ventilation strategy on oxygenation, DO_2_, stress, strain and regional air distribution may differ between different ventilation settings. We titrated PEEP according to the lowest *E*_stat,RS_ to minimize *P*_driv_, the only ventilation parameter during ECMO that was independently associated with mortality in a recent analysis [7].

Finally, the calculation of DO_2_ is based on stroke volume measurements with transpulmonary thermodilution and not echocardiography which is commonly defined as gold standard [54]. Although SV measurement with transpulmonary thermodilution has recently been shown not to be affected by ECMO blood flow [55 we cannot exclude a systematical error of CO measurement.

## Conclusions

In patients with severe ARDS managed with ECMO, apneic ventilation decreased oxygenation but preserved DO_2_ due to an increase in CO. Ultraprotective ventilation considerably increased stress, strain and mechanical power. As the optimal ventilation strategy in ECMO patients is unclear, apneic ventilation might be considered as one of the options in the initial phase of ECMO treatment in severe ARDS patients to facilitate lung rest and prevent VILI.

## Supplementary Information

**Additional file 1. **Inclusion criteria, exclusion criteria, criteria for discontinuation of the study. **Fig. S1.** Schematic flow chart of the study design. Ventilator management. Calculation of respiratory system mechanics. Computed tomography assessment.

## Data Availability

The data sets used and analyzed during the current study are available from the corresponding author on reasonable request.

## Abbreviations

APACHE II: Acute physiology and chronic health evaluation II score
ARDS: Acute respiratory distress syndrome
CO: Cardiac output
CT: Computed tomography
CVP: Central venous pressure
DO_2_: Oxygen delivery
ECMO: Extracorporeal membrane oxygenation
ELSO: Extracorporeal life support organisation
*E*_stat,RS_: Static elastance of the respiratory system
*E*_stat,L_: Static elastance of the lung
*E*_stat,CW_: Static elastance of the chest wall
FiO_2_: Fraction of inspired oxygen
Hb: Hemoglobin
HR: Heart rate
IBW: Ideal body weight
ICU: Intensive care unit
MAP: Mean arterial pressure
MV: Mechanical ventilation
PaCO_2_: Arterial partial pressure of carbon dioxide
pH: Negative logarithm of the molar concentration of dissolved hydronium ions
PaO_2_: Arterial partial pressure of oxygen
*P*_driv_: Driving pressure
PEEP: Positive end-expiratory pressure
*P*_mean_: Mean airway pressure
*P*_peak_: End-inspiratory peak pressure
*P*_plat_: End-inspiratory plateau pressure
PRESERVE: Predicting Death for Severe ARDS on vv-ECMO score
RESP: Respiratory ECMO survival prediction score
RR: Respiratory rate
SAPS II: Simplified acute physiology score II
SaO_2_: Arterial oxygen saturation
SOFA: Sequential Organ Failure Assessment
VCV: Volume-controlled ventilation
VILI: Ventilator-induced lung injury
*V*_T_: Tidal volume
ZEEP: Zero end-expiratory pressure
