# Supplementary material for: Ultraprotective versus apneic ventilation in acute respiratory distress syndrome patients with extracorporeal membrane oxygenation: a physiological study
Source: J Intensive Care. 2022 Mar 7;10:12. doi: 10.1186/s40560-022-00604-9 (PMC8900404; doi:10.1186/s40560-022-00604-9)
Supplement: Supplementary file 1 — Additional file 1. Inclusion criteria, exclusion criteria, criteria for discontinuation of the study. Fig. S1. Schematic flow chart of the study design. Ventilator management. Calculation of respiratory system mechanics. Computed tomography assessment. [file 40560_2022_604_MOESM1_ESM.docx]

**Protective *versus* apneic ventilation in acute respiratory distress syndrome patients with extracorporeal membrane oxygenation: a physiological study**

Peter T Graf^1^; Christoph Boesing^1^; Isabel Brumm^1^; Jonas Biehler^2,3^; Kei Wieland Müller^2,3^; Manfred Thiel^1^; Paolo Pelosi^4,5^; Patricia RM Rocco^6^; Thomas Luecke^1^; Joerg Krebs^1^

^1^ Department of Anesthesiology and Critical Care Medicine, University Medical Centre Mannheim, Medical Faculty Mannheim of the University of Heidelberg, Mannheim, Germany, Theodor-Kutzer Ufer 1-3, 68167 Mannheim, Germany

^2^ Institute for Computational Mechanics, Technical University Munich, Boltzmannstraße 15, 85748 Garching

^3^ Ebenbuild GmbH Schinkelstrasse 44, 80805 Munich

^4^ Department of Surgical Sciences and Integrated Diagnostics, University of Genoa, Viale Benedetto XV 16, Genoa, Italy

^5^ Anesthesiology and Critical Carel, San Martino Policlinico Hospital, IRCCS for Oncology and Neurosciences, Genoa, Italy

^6^ Laboratory of Pulmonary Investigation, Carlos Chagas Filho Institute of Biophysics, Federal University of Rio de Janeiro, Centro de Ciências da Saúde, Avenida Carlos Chagas Filho, 373, Bloco G-014, Ilha do Fundão, Rio de Janeiro, Brazil

Peter T Graf and Christoph Boesing equally contributed to this work

**E-mail adresses:**

Peter T Graf: Tobias.Graf@umm.de

Christoph Boesing: Christoph.Boesing@umm.de

Isabel Brumm: Isabel.Brumm@umm.de

Jonas Biehler: biehler@lnm.mw.tum.de

Kei Wieland Müller: kei.mueller@tum.de

Manfred Thiel: manfred.thiel@umm.de

Paolo Pelosi: ppelosi@hotmail.com

Patricia RM Rocco: prmrocco@biof.ufrj.br

Thomas Luecke: thomas.luecke@medma.uni-heidelberg.de

# Table of Contents

# Inclusion criteria, exclusion criteria, criteria for discontinuation of the study

# Supplemental Figure 1: Schematic flow chart of the study design

# Ventilator management

# Calculation of respiratory system mechanics

# Computed tomography assessment

# Inclusion criteria, exclusion criteria, criteria for discontinuation of the study

# Inclusion criteria:

# written informed consent from substitute decision-makers

# severe acute respiratory distress syndrome according to the Berlin definition ([1](#_ENREF_1))

# veno-venous extracorporeal membrane oxygenation treatment

# Exclusion criteria:

# age younger than 18 years

# pregnancy

# end-stage chronic organ failure

# inherited cardiac malformations

# severe head injury

# hemodynamic instability (mean arterial pressure <65 mmHg despite sufficient intravascular volume and norepinephrine administration, cardiac index <2.0 l/min/m^2^ despite sufficient intravascular volume and dobutamine administration)

# Criteria for discontinuation of the study:

# new onset of hemodynamic instability (mean arterial pressure <65 mmHg despite sufficient intravascular volume and norepinephrine administration, cardiac index <2.0 l/min/m^2^ despite sufficient intravascular volume and dobutamine administration)

# Oxygen delivery (DO_2_) < 400ml/min and new onset signs of inadequate DO_2_ (electrocardiogram changes, signs of hypoperfusion or shock, oxygen extraction ratio >50%, rise in serum lactate) ([2](#_ENREF_2))

# Figure S1: Schematic flow chart of the study design

#



PEEP, *positive end-expiratory pressure*; FiO_2_, *fraction of inspired oxygen*, CT, *computed tomography*

# Ventilator Management

**Ultraprotective ventilation**

| Ventilator mode | FiO_2_ | RR | V_T_ | PEEP | I:E |
| --- | --- | --- | --- | --- | --- |
| VCV | 21% | 12/min | 3 ml/kg IBW | lowest E_stat,RS_ | 1:1 |
| VCV | 50% | 12/min | 3 ml/kg IBW | lowest E_stat,RS_ | 1:1 |
| VCV | 90% | 12/min | 3 ml/kg IBW | lowest E_stat,RS_ | 1:1 |

**Apneic ventilation**

| Ventilator mode | FiO_2_ | RR | V_T_ | PEEP | I:E |
| --- | --- | --- | --- | --- | --- |
| CPAP | 21% | / | / | lowest E_stat,RS_ | / |
| CPAP | 50% | / | / | lowest E_stat,RS_ | / |
| CPAP | 90% | / | / | lowest E_stat,RS_ | / |

VCV, *volume-controlled ventilation*; FiO_2_, *fraction of inspired oxygen*; RR, *respiratory rate*; V_T_, *tidal volume*; IBW, *ideal body weight*; PEEP, *positive end-expiratory pressure*; E_stat,RS_, *static elastance of the respiratory system*; I:E, *inspiration-to-expiration ratio*; CPAP, *continuous positive airway pressure*

# During apneic ventilation, backup ventilation was deactivated on the ventilator (Engström Carescape™, GE Healthcare, Munich, Germany) to avoid triggering of alarms.

# Calculation of respiratory system mechanics and delivery of oxygen

Driving pressure (P_driv_) = end-inspiratory plateau pressure (P_plat_) – PEEP

difference between end-inspiratory and end-expiratory esophageal pressure (∆Pes) = end-inspiratory esophageal pressure – end-expiratory esophageal pressure

Static elastance of the respiratory system (E_stat,RS_) = P_driv_/tidal volume (V_T_)

Static elastance of the lung (E_stat,L_) = (P_driv_ - ∆Pes)/V_T_

Static elastance of the chest wall (E_stat,CW_) = ∆Pes/V_T_

Stress = P_plat_ – end-inspiratory esophageal pressure

Mechanical power = 0.098 x V_T_ x RR x (end-inspiratory peak pressure (P_peak_) – 0.5 x P_driv_) according to Serpa Neto et al. ([3](#_ENREF_3))

DO_2_ = Cardiac output x 1.34 x hemoglobin x arterial oxygen saturation + (0.003 x arterial partial pressure of oxygen)

# Computed tomography assessment

Lung parenchyma was classified as non-aerated (-100 to +100 hounsfield units), poorly inflated (-500 to -100 hounsfield units), normally inflated (**-**501 to -900 hounsfield units) and overinflated (<-900 hounsfield units) in the computed tomography (CT) scans. The lung was segmented into a non-dependent and dependent compartment along a horizontal plane through the tracheal bifurcation. Lung air volumes were calculated as the cumulative air volume of poorly, normally and overinflated lung. Voxel tissue weight was calculated as (1– (Voxel density−1000)) x Voxel volume. Lung weight was calculated as the cumulative voxel weight ([4](#_ENREF_4)). Percentage of lung recruitment due to the application of PEEP was calculated as (nonaerated lung tissue at zero end-expiratory pressure (ZEEP) - nonaerated lung tissue at PEEP)/total lung weight at ZEEP) x 100. Percentage of lung recruitment due to the application of a tidal volume was calculated as (nonaerated lung tissue at PEEP – nonaerated lung tissue at P_plat_) / total lung weight at PEEP) x 100 ([5](#_ENREF_5)). Total strain was calculated as end-inspiratory lung air volume divided by lung air volume at ZEEP. Static strain was calculated as end-expiratory lung air volume at PEEP divided by end-expiratory lung air volume at ZEEP. Dynamic strain was calculated as total strain – static strain.

**Abbreviations:**

CT computed tomography

DO_2_ delivery of oxygen

E_stat,RS_ static elastance of the respiratory system

E_stat,L_ static elastance of the lung

E_stat,CW_ static elastance of the chest wall

FiO_2_ fraction of inspired oxygen

IBW ideal body weight

P_driv_ driving pressure

∆Pes difference between end-inspiratory and end-expiratory esophageal pressure

PEEP positive end-expiratory pressure

P_peak_ end-inspiratory peak pressure

P_plat_ end-inspiratory plateau pressure

RR respiratory rate

VCV volume-controlled ventilation

V_T_ tidal volume

ZEEP zero end-expiratory pressure

# References

1. ARDS Definition Task Force, Ranieri VM, Rubenfeld GD, Thompson BT, Ferguson ND, Caldwell E, et al. Acute respiratory distress syndrome: the Berlin Definition. JAMA. 2012;307(23):2526-33.

2. Schoettler JJ, Kirschning T, Hagmann M, Hahn B, Fairley AM, Centner FS, et al. Maintaining oxygen delivery is crucial to prevent intestinal ischemia in critical ill patients. PLoS One. 2021;16(7):e0254352.

3. Serpa Neto A, Deliberato RO, Johnson AEW, Bos LD, Amorim P, Pereira SM, et al. Mechanical power of ventilation is associated with mortality in critically ill patients: an analysis of patients in two observational cohorts. Intensive Care Med. 2018;44(11):1914-22.

4. Protti A, Iapichino GE, Milesi M, Melis V, Pugni P, Comini B, et al. Validation of computed tomography for measuring lung weight. Intensive Care Med Exp. 2014;2(1):31.

5. Ball L, Robba C, Maiello L, Herrmann J, Gerard SE, Xin Y, et al. Computed tomography assessment of PEEP-induced alveolar recruitment in patients with severe COVID-19 pneumonia. Critical care (London, England). 2021;25(1):81.
